# Features of the course of affective diseases in hyperthymic persons

**DOI:** 10.1192/j.eurpsy.2023.1447

**Published:** 2023-07-19

**Authors:** A. Barkhatova, A. Churkina, M. Bolgov

**Affiliations:** Department of endogenous mental disorders and affective states, Mental health research center, Moscow, Russian Federation

## Abstract

**Introduction:**

Hyperthymia was studied by many eminent psychiatrists considering it mainly within the framework of the subsyndromic course of affective disorders (Akiskal H.S., Angst J., Benazzi F.). Statistical data on the prevalence of hyperthymic personalities are scarce, which is associated with rare requests for help and diagnosis of this condition within the framework of character traits. Given the above, the study of the psychopathological and dynamic features of hyperthymia seems to be very relevant, since previously hyperthymic characterological features were not always taken into account in the diagnosis of the disease, the selection of drugs and the prediction of remission.

**Objectives:**

Determination of the clinical features of the course of an affective disease in patients with hyperthymic features.

**Methods:**

The sample consisted of, 48 patients (39 women, 9 men) who were on inpatient or outpatient treatment at the clinic since 2019 to 2022. Patients were examined by clinical-psychopathological, clinical-anamnestic methods due to the presence of a phase affective state.

**Results:**

The subjects were diagnosed with two diseases - bipolar affective disorder and cyclothymia. There were 2 variants of affective phases - affective-mixed and “double” mania. In bipolar disorder, there were anxiety-hypochondriacal depressions (10 patients, 38.5%), polymorphic depressions (4 patients, 15.4%), asthenic hypomania (5 patients, 19.2%), hypochondriacal manias (3 patients, 11. 5%), alternating states (4 patients, 15.4%), “double” manias (8 patients, 23.5%). In cyclothymia, anxiety-hypochondriac depression (8 patients, 57.1%), somatoform decompensation (2 patients, 14.3%) and asthenic hypomania (4 patients, 28.6%) were observed.

**Conclusions:**

Thus, bipolar disorder was the most common among hyperthymic individuals. Anxiety-hypochondriacal depression prevailed in both nosologies. Manic and hypomanic states were most often observed in patients with bipolar disorder (“double” manias - exclusively in bipolar disorder).

**Disclosure of Interest:**

None Declared

